# Consensus Statement on Full-Arch Implant Rehabilitations: Evidence-Based Recommendations from the Italian Consensus Conference

**DOI:** 10.3390/jcm15103695

**Published:** 2026-05-11

**Authors:** Biagio Rapone, Elisabetta Ferrara, Filippo Tomarelli, Giuseppe Giovannico, Christian Bacci, Grazieli Dalmaschio, Massimiliano Novello, Antonio Andrisani, Giuseppe De Caro, Elena Fontanella, Paolo Dal Maso, Alessandro Buso, Alberto Ragagnin, Marco Ronda, Fabio Bernardello, Carlo Baroncini, Salvatore Galentino, Danilo Azzolini, Nicola Barion, Paolo Bozzoli, Vittorio Giannelli, Alessandro Mazzotta, Filippo Muratore, Maurizio Grande, Costantino Giagnorio, Caterina Nardi, Gilberto Gallelli, Luca Erboso, Maurizio De Francesco

**Affiliations:** 1 Interdisciplinary Department of Medicine, “Aldo Moro” University of Bari, 70121 Bari, Italy; 2 Department of Human Sciences, Law, and Economics, Telematic University “Leonardo Da Vinci”, UNIDAV, Torrevecchia Teatina, 66100 Chieti, Italy; elisabetta.ferrara@unidav.it; 3 Private Practice, 00197 Rome, Italynovello.massimiliano@libero.it (M.N.); dr.antonioandrisani@gmail.com (A.A.); fontanellaelena@gmail.com (E.F.); dottbuso@libero.it (A.B.); alberto.ragagnin@tiscali.it (A.R.); info@studiomarcoronda.it (M.R.); fabio.tredici@libero.it (F.B.); salvatoregalentino@gmail.com (S.G.); nicola.barion@gmail.com (N.B.); p.bozzoli@eliteodontoiatrica.it (P.B.); dott.vittoriogiannelli@gmail.com (V.G.); alessmazzotta@gmail.com (A.M.); filippo.muratore@gmail.com (F.M.); maurizio.grande@inwind.it (M.G.); lucaerboso@gmail.com (L.E.); 4 Department of Medicine and Health Science “Vincenzo Tiberio”, University of Molise, 86100 Campobasso, Italy; 5 Department of Neurosciences, University of Padua, 35122 Padua, Italygrazieli@hotmail.it (G.D.); studio.dottdefrancesco@gmail.com (M.D.F.); 6 Self-Employed Owner of a Dental Laboratory, 31044 Montebelluna (TV), Italy; decarogiuseppe4@gmail.com (G.D.C.); artedental@hotmail.it (P.D.M.); carlobaroncini@me.com (C.B.); dazzolini.vr@gmail.com (D.A.); g.gallelli@bio3d.it (G.G.); 7 “Policlinico Foggia” University Hospital, 71122 Foggia, Italy; costantino.giagnorio@gmail.com

**Keywords:** dental implants, full-arch rehabilitation, All-on-4, consensus statement, peri-implantitis, guided bone regeneration, monolithic zirconia, MRONJ, maintenance protocol, GRADE, Delphi method

## Abstract

Full-arch implant-supported rehabilitations are widely recognized as an effective treatment option for edentulous patients. Nevertheless, clinical decision-making regarding patient selection, surgical planning, prosthetic material choice, and long-term maintenance protocols remains heterogeneous and requires structured evidence-based guidance. A modified Delphi consensus process was conducted involving 29 experts during the Italian Consensus Conference. A systematic literature review covering the period 2015–2024 was performed, and the certainty of evidence was assessed using the Grading of Recommendations Assessment, Development and Evaluation (GRADE) framework. Consensus was predefined as ≥90% agreement. Seven evidence-based consensus statements were developed addressing: (1) periodontal risk assessment using validated tools; (2) guided bone regeneration outcomes with technique-specific indications; (3) comparative survival of four versus six implants in mandibular full-arch rehabilitations; (4) equivalence of tilted and axial implant configurations; (5) prosthetic material selection, with monolithic zirconia showing high survival; (6) risk-stratified supportive maintenance protocols associated with a reduction in peri-implantitis incidence; and (7) systemic risk stratification, including absolute and relative contraindications, medication-related osteonecrosis of the jaw (MRONJ) risk management, and perioperative antibiotic prophylaxis.

## 1. Introduction

Full-arch implant-supported rehabilitations are widely adopted for the management of complete edentulism and are associated with improvements in oral function and patient-reported outcomes when compared with conventional removable prostheses [1,2]. Over the last decades, clinical protocols have progressively shifted toward simplified, standardized approaches that aim to reduce surgical morbidity and overall treatment time while maintaining long-term predictability. Within this context, the All-on-4^®^ concept has been extensively disseminated as a clinically relevant protocol, and long-term observational data have been reported for full-arch immediate-function rehabilitations [3]. Despite broad clinical adoption, evidence supporting full-arch rehabilitation strategies remains heterogeneous, and several decision points continue to generate variability across clinicians and centers. These include the optimal number of implants (particularly in complete-arch fixed prostheses), the selection of axial versus tilted implant configurations, and the appropriate indications for adjunctive surgical procedures in compromised sites. Recent systematic evidence suggests that, in the maxilla, four- versus six-implant designs may show comparable survival outcomes, although differences in marginal bone loss have been reported across groups and study designs [4]. Comparable findings have also emerged for mandibular full-arch rehabilitations, as detailed in Statement 3. Similarly, the use of tilted posterior implants to avoid anatomical structures and reduce cantilevers remains a key topic. A recent umbrella review with meta-analysis reported no significant differences in implant failure between tilted and axial implants supporting full-arch prostheses, while small but statistically significant differences in marginal bone loss may emerge at longer follow-up intervals in some datasets [5]. From a prosthetic standpoint, material selection has become increasingly central to long-term outcomes. Updated evidence indicates that full-arch implant-supported monolithic zirconia prostheses exhibit high short-term success in systematic reviews, though the authors consistently highlight the need for stronger long-term, high-evidence studies [6]. Recent clinical data on monolithic zirconia full-arch prostheses also report high survival over mid-term follow-up, supporting their use while underscoring the relevance of technical complication profiles and design parameters [7]. In parallel, biological complications—particularly peri-implant diseases—represent a major determinant of long-term success and are strongly influenced by preventive strategies and supportive care. Contemporary guideline and consensus documents emphasize structured, risk-informed supportive peri-implant care as a cornerstone of prevention and management [8,9]. Observational evidence in patients not enrolled in regular supportive therapy reports clinically relevant peri-implantitis prevalence ranges and identifies patient- and site-related risk indicators, reinforcing the need for maintenance protocols tailored to individual risk profiles [10]. Therefore, the present consensus statement was developed to provide structured, evidence-based recommendations for full-arch implant rehabilitations, addressing key surgical, prosthetic, and maintenance-related decisions. Recommendations were formulated through a formal consensus methodology and supported by systematic evidence appraisal according to AGREE II principles and the GRADE framework for certainty of evidence and strength of recommendations [11,12].

## 2. Materials and Methods

### 2.1. Consensus Panel Composition

The expert panel was established according to predefined eligibility criteria to ensure appropriate clinical expertise, scientific competence, and methodological balance. Selection criteria included:

(1) Documented clinical and/or academic experience of at least 10 years in implant dentistry and full-arch rehabilitation;
(2) Authorship of peer-reviewed publications within the last 10 years;
(3) Active membership in relevant national and international scientific societies;
(4) Geographic and institutional representativeness;
(5) Absence of relevant conflicts of interest;
(6) Commitment to active participation throughout the consensus process.

The final panel consisted of 29 experts, including 20 implant surgeons/prosthodontists, 4 academic researchers, 1 dental hygienist with expertise in periodontal medicine, and 5 dental technicians, reflecting a multidisciplinary approach encompassing surgical, prosthetic, and preventive perspectives.

### 2.2. Literature Search and GRADE Assessment

A comprehensive literature review was conducted to identify the most relevant evidence on full-arch implant-supported rehabilitations. Electronic databases including PubMed/MEDLINE, Cochrane Central Register of Controlled Trials (CENTRAL), Embase, and Scopus were screened for studies published between January 2015 and January 2026. Priority was given to systematic reviews with meta-analysis, randomized controlled trials, and high-quality prospective cohort studies. When recent syntheses were unavailable, well-designed retrospective studies and narrative reviews were also considered. The certainty of evidence was evaluated using the Grading of Recommendations Assessment, Development and Evaluation (GRADE) framework, categorizing evidence as High, Moderate, Low, or Very Low. The strength of recommendations was classified as Strong or Conditional, in accordance with established guideline methodology [11,12].

### 2.3. Consensus Process

Consensus development was conducted using a modified Delphi methodology, structured into three iterative voting rounds to achieve standardized agreement among panel members:

- Round 1: electronic pre-conference voting on draft statements;
- Round 2: in-person discussion and second-round voting during the Italian Consensus Conference (7–8 February 2026; Falcade, Italy);
- Round 3: post-conference final electronic voting on revised statements.

The Consensus Conference titled “Digital Full-Arch State of the Art: Clinical Consensus” was held on 7–8 February 2026, in Falcade (Belluno, Italy) and was organized by the Associazione “DOD—Digital Operative Dentistry”, of which the authors of the present article are members.

All 29 panel members participated in each voting round (100% response rate). Agreement was quantified using a 9-point Likert scale. Consensus was predefined as ≥90% of participants scoring each statement within the 7–9 range, consistent with established Delphi consensus thresholds. Following Round 2 discussion, minor wording refinements were incorporated to improve clarity and clinical applicability; no substantive changes to the core recommendations were required. All six consensus statements achieved the predefined agreement threshold in the final round, indicating a high level of expert convergence across the evaluated clinical domains (Table 1). The interval between the in-person consensus conference (February 2025) and manuscript submission reflects the time required to complete Rounds 2 and 3 of the Delphi process, to perform structured GRADE-based evidence appraisal for each statement, to conduct the supplementary literature search as detailed in Section 2.2, and to achieve internal panel approval of the final text. This timeline is consistent with comparable consensus publication processes in periodontology and implant dentistry, including the EFP S3 guideline on stage I–III periodontitis (workshop held in November 2019, published July 2020) and the AO/AAP Consensus on peri-implant diseases (workshop held in 2023, published in 2025).

Validated risk assessment systems include:

- The Periodontal Risk Assessment (PRA) model [13].
- The Staging and Grading classification framework from the 2017 World Workshop [14]:

Certainty of Evidence: High.

Strength of Recommendation: Strong.

Consensus Agreement: 96%.

### 2.4. Rationale and Clinical Implications

Patients with a history of periodontitis present a significantly increased susceptibility to biological complications following implant placement. Evidence demonstrates an approximately 4-fold increased risk of peri-implantitis in periodontal patients (RR = 4.09; 95% CI: 1.93–8.58) [15]. However, when periodontal infection is adequately controlled and patients adhere to an individualized supportive periodontal care program, long-term implant outcomes remain favourable. In a 20-year prospective cohort study, Roccuzzo et al. reported an overall implant survival rate of 93%, with no significantly increased odds of implant loss in periodontally compromised patients compliant with supportive care, whereas non-compliant patients exhibited a markedly higher risk of implant loss (OR = 14.59; 95% CI: 1.30–164.29) [16]. Therefore, careful stabilization and long-term supportive care are mandatory to minimize biological complications:

- Pre-operative stabilization of periodontal tissues;
- Strict elimination of residual inflammation;
- Risk-based supportive care scheduling;
- Long-term monitoring of peri-implant parameters.

This approach is essential to minimize peri-implant disease risk and to ensure predictable surgical and prosthetic outcomes.

#### Operational Criteria for Periodontal Stability Assessment Prior to Full-Arch Rehabilitation

The six-month periodontal stability criterion requires explicit operational thresholds, which the panel has formalised according to the pre-implant dentition status of the candidate.

In partially edentulous candidates with residual dentition, periodontal stability is operationally defined, in accordance with the European Federation of Periodontology (EFP) S3 clinical practice guideline for stage I–III periodontitis [17], as the simultaneous fulfilment, for a minimum of six months following completion of active periodontal therapy, of the following four criteria: full-mouth bleeding score (FMBS) < 15%; full-mouth plaque score (FMPS) < 20%; absence of residual sites with probing depth ≥ 5 mm associated with bleeding on probing; and absence of radiographic progression of alveolar bone loss on sequential periapical or panoramic radiographs. Failure to meet all four criteria simultaneously requires continuation of periodontal therapy and deferral of implant surgery.

The concept of periodontal stability cannot be applied stricto sensu to fully edentulous patients. In this subgroup, the panel proposes the operational concept of biological readiness for implant placement, defined as the simultaneous presence of: (i) control of behavioural and systemic risk factors, including smoking cessation or verified reduction to ≤10 cigarettes/day for at least three months, glycaemic control with HbA1c < 7.5% in diabetic patients, and appropriate management of medications affecting bone metabolism; (ii) a structured anamnesis of the cause of tooth loss, with particular attention to a documented history of advanced periodontitis, which should be interpreted as indicative of a progression-prone host response that may extend to peri-implant tissues; and (iii) assessment of residual mucosal phenotype, including keratinised tissue width at prospective implant sites, given the documented association between reduced keratinised mucosa and peri-implant inflammation [16,18].

Full individual predictive accuracy for peri-implant disease is not achievable with currently available tools, and this constitutes a fundamental clinical caveat that must be communicated to the patient during informed consent. Structured susceptibility profiling nevertheless improves the identification of patients requiring closer surveillance. The panel endorses the integrated use of: (1) the Periodontal Risk Assessment (PRA) model [13]; (2) the 2017/2018 Staging and Grading framework [14], with particular attention to Grade C as a marker of progression-prone phenotype; and (3) the Implant Disease Risk Assessment (IDRA) [19], an octagonal diagnostic framework developed specifically to estimate peri-implantitis risk through eight weighted vectors (history of periodontitis, FMBS, number of sites with PD ≥ 5 mm, ratio of bone loss to patient age, Staging/Grading category, compliance with supportive therapy, distance from the restorative margin to the marginal bone crest, and prosthesis-related cleanability). Retrospective validation in patients with treated periodontitis and implant-supported fixed dental prostheses has demonstrated a significant association between high IDRA classification and peri-implantitis development at five-year follow-up [20].

Periodontal stability assessment and peri-implantitis susceptibility profiling operate at different decisional stages: the former determines whether surgery should proceed, the latter calibrates the intensity and frequency of long-term supportive care (Statement 6, Table 2).

Clinical Take-Home Messages.

- Document periodontal stability for candidates: FMBS < 15%, FMPS < 20%, no PD ≥ 5 mm with BoP, no radiographic progression.
- For fully edentulous candidates, verify biological readiness: smoking cessation or reduction to ≤10 cigarettes/day, HbA1c < 7.5% in diabetic patients, documented prior compliance with supportive care.
- Apply PRA, 2017/2018 Staging and Grading, and IDRA in combination for peri-implantitis susceptibility profiling.
- Communicate explicitly to the patient that no current risk tool offers full predictive accuracy; this is part of the informed consent process.

## 3. Results

### 3.1. Statement 2: Guided Bone Regeneration

Implants placed in sites treated with guided bone regeneration (GBR) may achieve survival outcomes comparable to those placed in native bone, although augmentation procedures are associated with increased surgical complexity and risk of post-operative complications. GBR should be performed only when prosthetically driven implant placement cannot be achieved in pristine bone and requires strict adherence to regenerative principles.

Certainty of Evidence: Moderate Strength of Recommendation: Strong Consensus Agreement: 90%.

Note: A strong recommendation was formulated despite moderate certainty of evidence, based on the consistency of findings across studies, the clear benefit-harm balance favoring selective GBR indication, and the high clinical relevance of avoiding unnecessary surgical morbidity.

#### 3.1.1. Rationale and Surgical Considerations

Systematic evidence indicates that implant placement in regenerated bone does not result in clinically relevant differences in implant survival compared with native bone. In a meta-analysis on lateral ridge augmentation, Elnayef et al. confirmed that implants placed in regenerated bone can perform similarly to those placed in non-augmented bone [21]. However, GBR remains a technique-sensitive procedure. Urban et al. emphasized that predictable regenerative outcomes depend on fundamental surgical principles, including primary tension-free wound closure, graft stability, space maintenance, and prevention of membrane exposure [22]. Complications such as membrane exposure and infection represent the most frequent adverse events and may compromise the volume of regenerated bone and long-term stability. Monje et al. highlighted that complication rates in GBR procedures are clinically relevant and must be carefully considered during treatment planning [23].

#### 3.1.2. Clinical Application

The panel emphasizes that GBR should be indicated only when prosthetically driven implant placement cannot be achieved in native bone and when patient-related risk factors (e.g., smoking, history of periodontitis, systemic conditions affecting wound healing) have been appropriately assessed [22,23]. Whenever possible, regenerative strategies should aim to minimize surgical morbidity, since membrane exposure and graft-related infections remain among the most frequent complications that may compromise regenerative outcomes [23].

#### 3.1.3. Surgical Implications for Full-Arch Cases

In full-arch rehabilitation, the indication for regenerative procedures should be carefully weighed against alternative strategies to reduce cantilever length and improve biomechanical load distribution. As detailed in Statement 4, these may include the use of tilted posterior implants to avoid anatomical structures, the engagement of available basal or cortical bone, or the placement of short implants in selected cases [24,25,26]. Therefore, GBR should be considered a predictable but technique-sensitive adjunct, requiring experienced surgical execution and strict post-operative monitoring to minimize complication risk [22,23].

#### 3.1.4. Timing of Bone Augmentation: Simultaneous Versus Staged Approach

The timing of guided bone regeneration in relation to implant placement is a critical determinant of clinical outcome. The panel distinguishes between one-stage (simultaneous) and two-stage (staged) approaches, with indications based on defect morphology and achievable primary stability. Simultaneous guided bone regeneration with implant placement is indicated when the residual bone volume allows achievement of an insertion torque ≥ 35 Ncm or an implant stability quotient (ISQ) ≥ 70, and when the peri-implant defect is limited to ≤2 mm in horizontal dimension without dehiscence extending beyond the apical third of the implant. Under these conditions, meta-analytic evidence supports implant survival outcomes comparable to those observed in native bone, with a small increase in marginal bone loss at mid-term follow-up [21]. The principal clinical advantage is the reduction in overall treatment duration and in the number of surgical interventions. Staged guided bone regeneration with delayed implant placement, typically after a healing period of six months, is indicated when horizontal defects exceed 2 mm, when peri-implant dehiscence would compromise primary stability, or in anatomically complex sites where simultaneous regeneration would increase the risk of membrane exposure and graft failure. The staged approach is associated with more predictable horizontal augmentation outcomes and a lower rate of regenerative complications in compromised sites [22,23]. In full-arch cases, regenerative procedures should be considered only after alternative biomechanical strategies have been evaluated, including tilted posterior implants, engagement of basal or cortical bone, and the use of short implants (Statement 4) [24,25,26]. When guided bone regeneration is indicated, the staged approach is generally preferred in patients with documented risk factors for wound healing impairment (smoking, uncontrolled diabetes, history of advanced periodontitis), whereas the simultaneous approach may be considered in low-risk patients with favourable defect morphology.

Clinical Take-Home Messages.

1. Indicate GBR only when prosthetically driven implant placement cannot be achieved in pristine bone and after alternative strategies (tilted implants, short implants, basal bone engagement) have been excluded.
2. Prefer the one-stage approach when residual bone allows insertion torque ≥ 35 Ncm or ISQ ≥ 70 and peri-implant defect ≤ 2 mm.
3. Prefer the two-stage approach (6-month healing) when defects exceed 2 mm, in compromised wound-healing profiles, or in anatomically complex sites.
4. Address patient-related risk factors (smoking, diabetes, history of advanced periodontitis) before regenerative surgery.

### 3.2. Statement 3: Number of Implants—Mandible

#### 3.2.1. Consensus Recommendation on Mandibular Implant Number

In mandibular full-arch implant-supported rehabilitations, four implants may be sufficient in the majority of standard clinical scenarios, with implant survival outcomes comparable to six-implant configurations. In a large retrospective cohort study of immediately loaded full-arch fixed complete dentures (943 patients; 5989 implants; mean follow-up 5.0 ± 3.2 years), mandibular 5-year cumulative implant survival rates were 98.6% for four-implant designs and 99.4% for six-implant designs, with no statistically significant difference (*p* = 0.136) [27]. Placement of six implants may be preferred in patients presenting increased biomechanical or anatomical risk (e.g., severe mandibular ridge atrophy, documented bruxism, high occlusal forces, systemic conditions potentially affecting healing).

Certainty of Evidence: High.

Strength of Recommendation: Strong.

Consensus Agreement: 94%.

#### 3.2.2. Rationale: Mandibular Bone Quality and Comparative Survival Data

The mandible generally provides favorable bone density and cortical anchorage, which may explain the high success rates observed with reduced implant numbers. As noted in the Introduction, comparable findings have been reported for maxillary rehabilitations [4], although anatomical differences warrant site-specific recommendations. The comparative retrospective cohort by Caramês et al. represents the largest controlled dataset currently available comparing four- versus six-implant mandibular configurations, and reported no clinically meaningful difference in implant survival at 5 years [27]. Prosthetic and technical complications were not evaluated in this study and were beyond its scope; therefore, the panel acknowledges that evidence on mechanical outcomes remains limited.

#### 3.2.3. Surgical Selection Criteria for Four-Implant Configuration

From a surgical planning perspective, four implants may be selected when:

- Adequate anterior mandibular bone volume is present;
- Implant distribution allows favorable anteroposterior spread;
- Cantilever length is minimized;
- Occlusal scheme is properly controlled prosthetically.

Conversely, a six-implant approach may be preferred when load distribution must be enhanced in high-risk patients, in order to improve biomechanical support and potentially reduce mechanical complications.

#### 3.2.4. Consensus Guidance on Mandibular Implant Number

Therefore, implant number selection in mandibular full-arch rehabilitation should not be based solely on protocol preference, but rather on:

- Anatomical availability;
- Functional loading conditions;
- Patient-specific risk stratification;
- Prosthetically driven implant positioning.

Four implants represent the baseline option in standard mandibular cases, while six implants remain indicated in complex or high-load scenarios.

#### 3.2.5. Implant Geometry and Biomechanical Considerations

Implant survival in four-versus-six-implant mandibular designs is conditioned not only by implant number but also by the geometric features of the individual fixtures, which together determine the total bone-to-implant contact (BIC) surface and the biomechanical distribution of occlusal load across the arch. For terminal implant positions in mandibular four-implant designs, the panel recommends a minimum implant length of 10 mm and a minimum diameter of 4 mm, corresponding to a cumulative peri-implant cylindrical surface sufficient to distribute functional loads without local bone overload. Four implants of 13 × 5 mm provide approximately 2.6-fold cumulative lateral surface, and therefore the potential osseointegrated BIC, of four implants of 8 × 3.5 mm: a difference with direct biomechanical consequences for full-arch prostheses subjected to cantilever and off-axial loading. When the recommended minimum geometry cannot be achieved due to residual ridge atrophy or anatomical limitations, a six-implant configuration with reduced individual fixture dimensions is biomechanically preferable to a four-implant configuration with undersized terminal implants. This position is consistent with the EAO Consensus on short implants [25] and with Cochrane evidence on implant interventions [24], both of which indicate that cumulative BIC and implant distribution, rather than absolute implant count, are the primary determinants of biomechanical stability in full-arch rehabilitation [26]. The panel therefore emphasises that the decision between four and six implants cannot be divorced from the concurrent decision on implant dimensions: a four-implant design with adequate fixture geometry and optimal anteroposterior spread is biomechanically equivalent to six implants of smaller dimensions, whereas a four-implant design with undersized terminal fixtures is not.

Clinical Take-Home Messages.

1. Select four implants as the baseline mandibular full-arch configuration when adequate anterior bone volume, favourable A-P spread, and controlled occlusal scheme are present.
2. Select six implants when definite bruxism, severe ridge atrophy, uncontrolled systemic comorbidities, or a full opposing fixed dentition is documented.
3. For terminal implants in four-implant mandibular designs, ensure a minimum length of 10 mm and minimum diameter of 4 mm.
4. When minimum individual fixture geometry cannot be achieved, a six-implant configuration is biomechanically preferable to a four-implant design with undersized terminal implants.

### 3.3. Statement 4: Tilted vs. Axial Implants

#### 3.3.1. Consensus Recommendation on Tilted Versus Axial Implant Configuration

In full-arch implant-supported rehabilitations, tilted posterior implants (30–45°) should be considered an acceptable alternative to axial implants, as they demonstrate equivalent clinical outcomes, with no clinically relevant differences in short-term marginal bone loss (MD = 0.00 mm; 95% CI −0.01 to 0.02; *p* = 0.75), although small differences may emerge at longer follow-up [5]. Tilting of posterior implants should be preferred when it enables:

- Avoidance of anatomical limitations (e.g., maxillary sinus, inferior alveolar nerve region).
- Posterior anchorage without advanced grafting procedures.
- Reduction of distal cantilever length through improved anteroposterior spread.

Certainty of Evidence: High. Strength of Recommendation: Strong. Consensus Agreement: 94%.

#### 3.3.2. Rationale: Biomechanical Behaviour of Tilted Implants

The primary surgical objective in full-arch rehabilitation is to achieve prosthetically driven implant positioning while minimizing surgical morbidity and biomechanical overload. As noted in Statement 2, tilted implants may represent a valid alternative to guided bone regeneration in anatomically challenging sites. Tilting posterior implants provides a predictable strategy to improve implant distribution and reduce cantilever forces, as demonstrated in finite element analyses by Bevilacqua et al. [28]. Systematic clinical evidence indicates that tilted implants exhibit survival outcomes comparable to axially placed implants. A meta-analysis comparing tilted versus axial implants found no statistically significant differences in implant failure rates or marginal bone loss between the two configurations [29]. Similarly, a systematic review focusing on edentulous maxillae rehabilitated with full-arch prostheses supported by tilted or axial implants reported equivalent implant survival and marginal bone stability at mid-term follow-up [30].

#### 3.3.3. Surgical and Prosthetic Prerequisites for Tilted Implant Placement

Tilted implants require strict adherence to surgical and prosthetic principles, including:

- Accurate three-dimensional prosthetically driven planning to ensure proper emergence profile and screw-access feasibility [31].
- Achievement of adequate primary stability, particularly in immediate function protocols [32].
- Control of implant angulation through appropriate abutment selection and passively fitting frameworks [24].
- Careful management of load distribution to minimize micromovement and crestal stress [28,29].

When these prerequisites are respected, tilted implants represent a technique-sensitive but reliable option, particularly in patients where grafting procedures would increase morbidity, treatment time, and complication risk [30,31].

#### 3.3.4. Consensus Guidance on Tilted Versus Axial Implant Selection

Therefore, axial placement remains appropriate when anatomy allows, but tilted implants should not be considered inferior. They may be strategically selected to optimize biomechanics and reduce the need for augmentation procedures, while maintaining equivalent implant survival and peri-implant bone stability [29,30,31].

#### 3.3.5. Computer-Aided Implant Surgery in Full-Arch Rehabilitation

Prosthetically driven implant placement in full-arch rehabilitation requires three-dimensional planning accuracy that freehand surgery cannot reliably achieve, particularly when tilted posterior implants and immediate loading are combined within a single workflow. The panel therefore considers computer-aided implant surgery (CAIS) an integral component of contemporary full-arch protocols, and recognises three complementary approaches [33,34]. Static computer-aided implant surgery (s-CAIS) uses a tooth-supported, mucosa-supported, or bone-supported surgical template fabricated from the digital plan. The most recent meta-analysis of clinical studies reports a mean linear deviation of 1.11 mm (95% CI 1.02 to 1.19) at the coronal entry point, 1.40 mm (95% CI 1.31 to 1.49) at the apex, and 3.51° (95% CI 3.27 to 3.75) of angular deviation [35]. Fully guided protocols demonstrate significantly higher accuracy than pilot-guided protocols and are considered the current gold standard for full-arch cases [31,36]. Dynamic computer-aided implant surgery (d-CAIS), also termed dynamic navigation, uses optical tracking of surgical instruments in real time against the preoperative plan. Accuracy is comparable to s-CAIS, with marginally better control of angular deviation and the added clinical advantage of intraoperative modification of the implant position when anatomical findings diverge from the preoperative plan [36]. Dynamic systems are particularly suited to full-arch immediate-loading workflows in which multiple tilted implants must be placed with strict anteroposterior distribution. Robotic computer-aided implant surgery (r-CAIS) represents the most recent technological evolution and reports the lowest deviations across all parameters (approximately 0.6 mm coronal, 0.7 mm apical, 1.6° angular) [37]. Clinical penetration of robotic systems in full-arch protocols remains limited, and the panel considers robotic CAIS a promising but evolving technology whose routine application awaits further validation in controlled clinical settings.

#### 3.3.6. Implant-Abutment Connection and Crestal Bone Preservation

The configuration of the implant-abutment interface exerts a measurable influence on long-term marginal bone stability, prosthetic complication rates, and the microbiological sealing of the peri-implant compartment. Three main categories of connection are in current clinical use: external hexagonal connections, internal flat-to-flat connections, and internal conical connections, the latter including Morse-taper and platform-switched designs. A network meta-analysis of randomised clinical trials comparing the three connection categories identified conical interfaces as the most effective in preserving peri-implant marginal bone, with significant differences relative to external hexagonal connections (P = 0.011) [37]. Consistent findings were reported in an earlier systematic review on the influence of the implant-abutment interface, which concluded that crestal bone levels are better maintained when internal connections, and particularly conical configurations, are adopted [38]. The largest quantitative synthesis to date, encompassing 45,347 implants from 270 studies, stratified marginal bone loss by connection type and follow-up interval: at mid-term follow-up (2 to 5 years), mean ΔMBL was 1.03 mm (95% CI 0.72 to 1.34) for external hex connections, 0.73 mm (95% CI 0.58 to 0.88) for wide-cone or flat-to-flat configurations, and 0.45 mm (95% CI 0.34 to 0.56) for narrow-cone (<45°) bone-level connections, confirming the superiority of narrow-cone internal conical interfaces in preserving crestal bone over time [39]. Conical connections reduce abutment micromovement under cyclic loading, minimise the implant-abutment microgap, and limit bacterial microleakage at the interface, with direct implications for the preservation of peri-implant health. Network meta-analytic evidence indicates that conical connections are also associated with a lower rate of prosthetic complications compared with external hexagonal designs (P = 0.038) [37]. Full-arch prostheses distribute substantial and often asymmetric occlusal loads across the implant-abutment interfaces, and are particularly exposed to the cumulative biological and mechanical effects of micromotion at the connection level. The panel therefore recommends that conical implant-abutment connections (Morse-taper or platform-switched) be preferred for full-arch implant-supported rehabilitations whenever the prosthetic envelope and component availability permit, and particularly in patients with documented biomechanical risk (bruxism, high occlusal load, full opposing natural dentition) or with elevated susceptibility to peri-implant disease.

#### 3.3.7. Cantilever Design: Quantitative Criteria and Contraindications

The clinical use of distal cantilever extensions in full-arch implant-supported prostheses requires explicit geometric and patient-specific criteria that extend beyond the general recommendation of minimising cantilever length. The panel formalises these criteria as follows. The distal cantilever should not exceed 1.5 times the anteroposterior (A-P) implant spread, defined as the linear distance between the most anterior implant and the line connecting the most distal implants on each side of the arch. This threshold derives from the biomechanical analyses by Bevilacqua et al. [28] and is widely adopted in the implant prosthodontic literature. In practical terms, cantilever extensions greater than 15 mm in the mandible and greater than 10 mm in the maxilla should be avoided, regardless of the calculated A-P spread. Absolute contraindications to distal cantilever extension include: definite sleep bruxism with a natural antagonist dentition (§3.4.4); insufficient prosthetic vertical dimension (<10 mm) when monolithic zirconia is selected, due to the risk of structural fracture under occlusal load; and an A-P implant spread < 5 mm, which compromises the biomechanical leverage required to support cantilever loading without excessive stress concentration at the distal implants. Relative contraindications, for which cantilever extension may be considered only after careful case-by-case evaluation, include: probable bruxism; a full opposing fixed implant-supported prosthesis, which increases the biomechanical load transmitted through the cantilever; a documented history of mechanical complications in previous implant-supported prostheses; and peri-implant phenotypes with elevated susceptibility to peri-implantitis (see Section 2.4), given the documented association between biomechanical overload and peri-implant marginal bone loss. When cantilever extension is not contraindicated but a longer distal span would improve occlusal scheme or prosthetic aesthetics, the placement of an additional distal implant, including through tilted configuration or the engagement of basal bone, should be preferred over extension of the cantilever (Statement 4; Section 3.1.4).

### 3.4. Statement 5: Prosthetic Materials

For full-arch implant-supported fixed rehabilitations, zirconia-based frameworks—particularly monolithic zirconia—show high prosthesis survival and low rates of major technical complications. An updated narrative review on zirconia-based implant-supported full-arch prostheses reported an average prosthesis survival of 97.23% over a mean follow-up of 49.7 months, with an average complication rate of 2.25% [33]. Compared with monolithic zirconia, veneered zirconia restorations are more frequently associated with chipping, with reported chipping rates ranging from 15% to 54% in the included evidence base [33].

Certainty of Evidence: High.

Strength of Recommendation: Strong.

Consensus Agreement: 91%.

#### 3.4.1. Rationale and Prosthetic–Biomechanical Considerations

Material selection influences long-term outcomes because technical complications increase maintenance needs and may require repair or replacement, with implications for supportive care protocols (see Statement 6). Recent syntheses indicate that zirconia-based full-arch prostheses—especially monolithic designs—achieve high survival with low major complication rates [33]. Consensus-oriented guidance in prosthodontics and implant dentistry also frames material choice within overall risk control and maintenance planning [34].

Monolithic zirconia provides:

- High fracture resistance.
- Reduced risk of veneering ceramic chipping.
- Favorable wear stability under occlusal load.
- Improved long-term structural integrity in complete-arch frameworks.

In contrast, veneered prostheses remain more susceptible to cohesive and adhesive ceramic fractures, particularly in high-load patients and in the presence of bruxism.

#### 3.4.2. Clinical and Prosthetic Implications

The panel emphasizes that prosthetic material selection should be integrated with biomechanical risk control, including:

- Ensuring adequate prosthetic space and contour.
- Achieving passive fit of the framework.
- Controlling occlusal scheme and cantilever extension.
- Identifying patients at increased overload risk (e.g., bruxism).

The panel acknowledges that current evidence on monolithic zirconia is predominantly based on short- to mid-term follow-up; long-term data (>10 years) remain limited and represent a priority for future research.

#### 3.4.3. Consensus Prosthetic Guidance

Therefore, monolithic zirconia should be considered the preferred restorative option for definitive full-arch implant-supported prostheses, particularly in cases requiring high structural durability and reduced technical complication rates. When technical complications occur, the clinical decision between chairside repair and complete prosthesis replacement should be guided by the type and extent of the complication, the integrity of the framework, and the patient’s overall treatment history. Minor chipping of monolithic zirconia may be managed by intraoral polishing or composite repair, whereas framework fractures or repeated debonding events necessitate prosthesis replacement with reassessment of the biomechanical design.

#### 3.4.4. Operational Assessment of Bruxism in Full-Arch Implant Candidates

Bruxism is increasingly recognised as a relevant biomechanical risk factor in full-arch implant-supported rehabilitation, particularly in relation to prosthetic complications, veneering ceramic fractures, and cumulative mechanical stress at the implant-abutment interface. The panel adopts the terminology and diagnostic grading defined by the international consensus on the assessment of bruxism [40] and updated in the most recent international expert consensus [41]. Accordingly, the terms sleep bruxism and awake bruxism are used throughout the present document, and the outdated umbrella term parafunction is not adopted. Diagnostic grading follows the consensus three-tier system, in which possible bruxism is based on a positive self-report only, probable bruxism is based on a positive clinical inspection with or without a positive self-report, and definite bruxism requires instrumental confirmation, typically by portable electromyography for awake bruxism and by polysomnography with audio-video recording for sleep bruxism. The multidimensional Standardised Tool for the Assessment of Bruxism (STAB) is recommended for structured clinical evaluation [42]. Clinical assessment prior to full-arch rehabilitation should include: a structured history incorporating validated self-report questionnaires; inspection for intraoral and extraoral signs (occlusal wear facets, tongue indentations, linea alba on the buccal mucosa, masseter hypertrophy, morning jaw stiffness); and, when clinical signs are equivocal or risk stratification remains uncertain, ambulatory surface electromyography. Polysomnography is reserved for severe cases or for patients with comorbid sleep-disordered breathing. The clinical consequences of bruxism for implant outcomes are supported by the most recent meta-analysis on this topic, which included 27 studies and 12,369 implants and reported a significantly increased risk of implant failure in probable bruxers compared with non-bruxers (odds ratio 2.189, 95% CI 1.337 to 3.583, *p* = 0.002) [43]. No statistically significant effect of follow-up duration on this association was observed, indicating that the increased failure risk persists over time rather than being limited to the early loading phase. In patients with definite bruxism, the panel recommends the following clinical protocol: selection of monolithic zirconia as the prosthetic material of choice (Statement 5); avoidance or strict limitation of distal cantilevers (§3.3.4); preference for a six-implant configuration over a four-implant design when anatomy permits (Statement 3); preference for conical implant-abutment connections (§3.3.6); prescription of a hard occlusal splint for nocturnal protection; and closer maintenance recall at three-month intervals (Statement 6, Table 2). The increased mechanical risk should be explicitly discussed with the patient during informed consent.

### 3.5. Statement 6: Maintenance Protocols (Surgical–Clinical Revision)

Systematic supportive maintenance programs are essential for long-term success in full-arch implant rehabilitations. Supportive therapy has been associated with a marked reduction in peri-implantitis incidence, with meta-analytic evidence suggesting an approximate 75% risk reduction in patients enrolled in structured maintenance protocols [44]. Maintenance recall intervals must be individualized according to patient-specific risk stratification, consistent with the periodontal Staging and Grading framework [14] and preventive consensus guidance [18,19]:

- Every 3–4 months in high-risk patients (Grade C).
- Every 4–6 months in moderate-risk patients (Grade B).
- Every 6 months in low-risk patients (Grade A).

Risk-based supportive visits should include peri-implant probing, bleeding assessment, professional biofilm removal, reinforcement of home-care, and occlusal monitoring [19,20]. Routine prosthesis removal is not recommended and should be performed only when clinically indicated, such as for diagnosis of inaccessible pathology or technical complications [18].

Certainty of Evidence: High.

Strength of Recommendation: Strong.

Consensus Agreement: 98%.

#### 3.5.1. Rationale and Clinical Considerations

Peri-implant diseases remain among the most significant biological complications affecting implant-supported full-arch prostheses. Their prevention relies primarily on early diagnosis, professional supportive care, and long-term patient adherence. As emphasized in Statement 1, patients with a history of periodontitis require particular attention due to their increased susceptibility to peri-implant diseases. Systematic maintenance has been shown to substantially reduce the incidence of peri-implantitis, reinforcing the necessity of structured follow-up protocols rather than episodic or symptom-driven visits. Non-compliance with supportive therapy represents the single most significant patient-related risk factor for peri-implant disease progression. As reported by Roccuzzo et al. [16], non-compliant patients exhibit a markedly higher risk of implant loss (OR = 14.59; 95% CI: 1.30–164.29) compared with compliant patients over a 20-year follow-up. The panel recommends that compliance status be reassessed at each supportive visit, that barriers to attendance be actively explored, and that patients demonstrating persistent non-compliance be reclassified to the high-risk maintenance category (Table 2) regardless of other risk indicators.

#### 3.5.2. Risk-Based Maintenance Strategy

The panel emphasizes that maintenance scheduling should be based on validated risk indicators, including:

- History of periodontitis (see Statement 1);
- Smoking habits;
- Systemic conditions affecting immune response or healing;
- Plaque control and patient compliance;
- Presence of bruxism or prosthetic overload;
- Peri-implant soft tissue status.

High-risk patients require closer monitoring to detect early inflammatory changes and prevent progression toward advanced peri-implant breakdown. Formal stratification of peri-implant disease risk is recommended using the Implant Disease Risk Assessment (IDRA) tool [19], which integrates eight weighted vectors (history of periodontitis, percentage of sites with bleeding on probing, number of teeth and implants with probing depth ≥ 5 mm, ratio of bone loss to patient age, 2017/2018 Staging and Grading category, compliance with supportive therapy, distance from the restorative margin to the marginal bone crest, and prosthesis-related cleanability) to classify patients into low, moderate, or high risk categories. The tool is openly available at perio-tools.com/idra and has been validated in a retrospective cohort of patients with treated periodontitis and implant-supported fixed dental prostheses, where high IDRA classification was associated with a higher prevalence of peri-implantitis at five-year follow-up [20]. For partially edentulous patients with residual dentition, IDRA should be used in combination with the Periodontal Risk Assessment (PRA) [13]. Risk-based recall intervals are defined as six months for low-risk patients, four months for moderate-risk patients, and three months for high-risk patients. Each supportive visit should include peri-implant probing at six sites per implant, assessment of bleeding on probing, full-mouth plaque score, professional biofilm removal with implant-safe instruments, reinforcement of individualised home care, and occlusal screening. Periapical or panoramic radiographs are recommended annually for low-risk patients, every 12 to 18 months for moderate-risk patients, and every 6 to 12 months for high-risk patients, with the latter group also receiving standardised photographic records at each visit. Intervention thresholds guide the escalation of supportive care within a phased treatment framework consistent with the EFP S3 clinical practice guideline on the prevention and treatment of peri-implant diseases [8]. At any implant site, a probing depth ≥ 5 mm associated with bleeding on probing triggers non-surgical mechanical debridement with implant-safe instruments, antiseptic adjunct, reinforcement of self-performed oral hygiene, and clinical re-evaluation at two months. Persistence of bleeding on probing at re-evaluation or progression to probing depth ≥ 6 mm indicates the need for adjunctive antimicrobial therapy and referral for surgical re-evaluation. Radiographic marginal bone loss of ≥ 2 mm beyond baseline remodelling at any stage escalates the protocol directly to surgical re-evaluation and, depending on the defect configuration, to reconstructive or resective therapy [45]. The complete decision matrix, including risk-specific recall intervals, mandatory assessments, and intervention thresholds, is summarized in Table 2.

#### 3.5.3. Clinical Application in Full-Arch Rehabilitation

Supportive therapy visits should include:

- Peri-implant probing and bleeding assessment;
- Evaluation of plaque and mucosal inflammation;
- Occlusal control and identification of overload;
- Professional biofilm removal with implant-safe instruments;
- Reinforcement of individualized home-care protocols.

Prosthesis removal should be reserved for situations such as:

- Suspected peri-implant pathology not accessible otherwise;
- Technical complications requiring intervention;
- Hygiene limitations due to prosthetic design.

#### 3.5.4. Consensus Clinical Guidance

Therefore, full-arch implant rehabilitation must be regarded as a long-term therapy requiring lifelong supportive care. Risk-stratified maintenance represents a cornerstone for reducing biological complications and ensuring sustainable implant and prosthetic survival.

### 3.6. Statement 7: Systemic Risk Stratification, Contraindications, and Perioperative Management

#### 3.6.1. Consensus Statement on Systemic Risk Stratificationt

Full-arch implant rehabilitation must be preceded by structured systemic risk stratification. Absolute and relative contraindications, perioperative antibiotic prophylaxis, and the specific risk of medication-related osteonecrosis of the jaw (MRONJ) must be systematically assessed and documented in every candidate, with the scope of treatment adapted accordingly.

Certainty of Evidence: Moderate. Strength of Recommendation: Strong. Consensus Agreement: 94%.

#### 3.6.2. Rationale

Candidates for full-arch implant rehabilitation are typically adults in the sixth to eighth decade of life, in whom polypharmacy and systemic comorbidities are prevalent. Evidence-based patient selection cannot rely on the single criterion of edentulism but requires a structured assessment of systemic health, pharmacological history, and perioperative risk. The panel agrees that three domains deserve explicit operational definition: absolute and relative contraindications, risk of medication-related osteonecrosis of the jaw (MRONJ), and antibiotic prophylaxis.

#### 3.6.3. Absolute and Relative Contraindications

The panel distinguishes absolute from relative contraindications, recognising that the former preclude implant placement regardless of other considerations, while the latter require case-by-case evaluation in a multidisciplinary context. Absolute contraindications to full-arch implant rehabilitation include: active and untreated malignancy of the oral or maxillofacial region; high-dose head and neck radiotherapy to the jaws, for which the risk of osteoradionecrosis is dose-dependent and increases substantially at cumulative doses exceeding 50 to 60 Gy, although no universally accepted threshold has been established; ongoing high-dose bone-modifying agent (HD-BMA) therapy for bone metastases, multiple myeloma, or giant cell bone tumour, when no drug holiday is medically feasible (see §3.6.4); acute severe immunosuppression (absolute neutrophil count < 1000/mm^3^, active chemotherapy cycle, solid organ transplantation within the preceding six months); uncontrolled bleeding disorders not amenable to correction; and severe uncontrolled mental health conditions precluding informed consent. Relative contraindications requiring multidisciplinary evaluation and individualised protocols include: uncontrolled diabetes mellitus (HbA1c ≥ 7.5%); current heavy smoking (>10 cigarettes/day); history of head and neck radiotherapy at lower cumulative doses or older than 12 months from completion; long-term low-dose bone-modifying agent (LD-BMA) therapy for osteoporosis; current antiangiogenic therapy without concurrent BMA; active autoimmune disease under immunosuppressive treatment; chronic corticosteroid therapy (prednisone-equivalent ≥ 10 mg/day for ≥3 months); and Sjögren syndrome or severe xerostomia of any cause. Advanced chronological age, in the absence of relevant comorbidities, is not a contraindication, and chronological age must be distinguished from biological status in clinical decision-making.

#### 3.6.4. MRONJ Risk Stratification and Management

The panel endorses the Italian position paper on MRONJ [46] as the reference framework for risk stratification in the Italian clinical setting, recognising its alignment with the American Association of Oral and Maxillofacial Surgeons (AAOMS) 2022 update [47]. Four categories of patients at increased MRONJ risk are recognised: patients with bone metastases or multiple myeloma receiving HD-BMA therapy; patients with breast or prostate cancer receiving LD-BMA therapy for cancer-treatment-induced bone loss (CTIBL); patients receiving denosumab for giant cell bone tumour; and patients receiving antiangiogenic therapy without concurrent BMA. Within the HD-BMA category, the Italian Society of Oral Pathology and Medicine (SIPMO)- Italian Society of Maxillofacial Surgery (SICMF) stratification further recognises R0 (therapy planned but not yet commenced), R+ (therapy ongoing, no additional systemic risk factors) and R++ (therapy ongoing with additional systemic risk factors, including antiangiogenic co-therapy). For patients in categories HD-BMA R+ and R++, the panel confirms that elective full-arch implant placement is generally contraindicated. For patients in category HD-BMA R0, the panel recommends that all indicated invasive dental procedures be completed and soft-tissue healing be confirmed prior to initiation of BMA therapy. For patients in category LD-BMA Rx (osteoporosis), implant placement is considered feasible provided that a structured informed consent process documents the low but non-quantifiable MRONJ risk, that local risk factors (periodontal disease, peri-implant infection, chronic periapical pathology) are eliminated prior to surgery, and that a specific peri-implant maintenance programme is established (see Statement 6). The panel emphasises that dental, periapical, periodontal, and peri-implant infections are the principal local risk factors for MRONJ. Accordingly, in patients receiving or scheduled to receive bone-modifying agents, the prevention of MRONJ begins with the comprehensive stabilisation of oral health before implant therapy, and with lifelong supportive peri-implant care delivered within a multidisciplinary model, as outlined for dental hygienists by the Italian expert position paper [48].

#### 3.6.5. Antibiotic Prophylaxis

The panel endorses the Consensus Report of the Italian Academy of Osseointegration [49] as the reference framework for antibiotic prophylaxis in implant surgery in the Italian setting, and integrates its recommendations with the most recent meta-analytic evidence [50,51]. For systemically healthy patients American Society of Anesthesiologists (ASA I or II) undergoing straightforward implant placement, the meta-analysis by Momand et al. [50] of seven randomised controlled trials with 1859 patients and 3014 implants reported no statistically significant reduction in early implant failure associated with antibiotic prophylaxis (RR 0.66, 95% CI 0.30 to 1.47). The network meta-analysis by Romandini et al. [51] identified a single pre-operative dose of 2 to 3 g of amoxicillin administered one hour before surgery as the most effective protocol for reducing implant failure. Full-arch rehabilitation, however, does not qualify as a straightforward case: it involves prolonged surgical time, extensive mucoperiosteal flap elevation, multiple implant sites, and, in many protocols, immediate loading. Accordingly, the panel recommends the following perioperative protocol: Pre-operative chlorhexidine 0.20% mouthwash for 60 s within two hours of surgery; single pre-operative dose of amoxicillin 2 to 3 g orally one hour before surgery (or clindamycin 600 mg in documented penicillin allergy). When simultaneous guided bone regeneration, sinus floor elevation, or extensive grafting is performed, post--operative antibiotic administration (amoxicillin 1 g every 12 h for up to six days) is indicated, consistent with the IAO consensus on graft-associated procedures [49]. For full-arch cases without concomitant regenerative procedures, the decision on post-operative antibiotic extension beyond the single pre-operative dose should be individualised, considering patient-specific risk factors, surgical duration, and intraoperative findings. Post-operative chlorhexidine 0.20% mouthwash twice daily for 14 days is recommended in all cases. Antibiotic stewardship principles apply: routine post-operative antibiotic courses exceeding six days are not supported by the evidence and are discouraged, and the selection of agents should be guided by local antimicrobial resistance patterns when available.

#### 3.6.6. Clinical Application

Systemic risk stratification should be performed at the first consultation and documented in the clinical record using a structured form that includes: active pharmacological therapy with specific attention to bone-modifying agents, antiangiogenics, immunosuppressants and corticosteroids; comorbidity profile including ASA classification; smoking status; periodontal phenotype; radiotherapy history; and allergy history. Multidisciplinary consultation must be obtained for any relative contraindication and is mandatory for patients in the LD-BMA Rx category. Informed consent for candidates with relative contraindications must explicitly document the modified risk profile discussed with the patient.

#### 3.6.7. Clinical Take-Home Messages

Perform structured systemic risk stratification in every full-arch implant candidate, using a written form that documents BMA and antiangiogenic therapy, comorbidities, ASA class, smoking, and radiotherapy history. Treat elective full-arch implant placement as generally contraindicated in patients receiving high-dose bone-modifying agents for oncological indications (HD-BMA R+ or R++). In candidates receiving low-dose BMA therapy for osteoporosis, place implants only after multidisciplinary consultation, elimination of local infectious risk factors, written informed consent documenting the non-quantifiable MRONJ risk, and enrolment in a dedicated maintenance protocol. Apply perioperative antibiotic prophylaxis based on the IAO 2021 framework [49]: chlorhexidine rinse before and after surgery, single pre-operative dose of amoxicillin 2 to 3 g (or clindamycin 600 mg in penicillin allergy). Extend post-operative coverage (up to 6 days) only when concomitant GBR or sinus elevation is performed.

## 4. Discussion

This Italian Consensus Conference provides seven evidence-based recommendations addressing key clinical decisions in full-arch implant rehabilitation. The high level of agreement achieved across all statements (90–98%) reflects both the maturity of the available evidence and the clinical relevance of the addressed topics. The modified Delphi methodology, combined with GRADE-based evidence appraisal, ensures methodological rigor consistent with contemporary guideline development standards [11,12]. The present recommendations align with and complement existing international consensus documents. Regarding periodontal risk assessment and maintenance (Statements 1 and 6), our findings are consistent with the EFP S3 clinical practice guideline, which states that “prevention of peri-implant diseases should commence when dental implants are planned, surgically placed and prosthetically loaded” and that “a supportive peri-implant care programme should be structured, including periodical assessment of peri-implant tissue health” [8]. Furthermore, the 2017 World Workshop on the Classification of Periodontal and Peri-Implant Diseases established that patients with peri-implant mucositis may develop peri-implantitis “especially in the absence of regular maintenance care” [18], reinforcing the rationale for our risk-stratified maintenance intervals. Regarding implant number and configuration (Statements 3 and 4), the 6th International Team for Implantology (ITI) Consensus Conference (2018) concluded that “there is no statistically significant difference in outcomes (implant and prosthesis survival) for full-arch fixed dental prostheses (FDPs) in the mandible supported by fewer than five implants when compared to five or more implants” [34]. The ITI Consensus also stated that “there is no statistically significant difference in primary outcomes (survival rates for implant and prosthesis) or secondary outcomes (peri-implant marginal bone loss, soft and hard tissue complications, prosthetic complications and patient-centred outcomes) for implants placed in an axial or in a tilted configuration when used to support full-arch FDPs,” based on 20 studies including 2 randomized controlled trials (RCTs) [34]. These findings directly support our Statements 3 and 4. The ITI clinical recommendation that “the anterior posterior implant distribution should be maximized for full-arch FDPs” and that “if anatomic limitations or prosthetic indications exist, the posterior implants can be intentionally tilted” [34] aligns with our consensus guidance. The recommendations of the present consensus are also coherent with the most recent international synthesis on peri-implant disease prevention and management, the AO/AAP Consensus on prevention and management of peri-implant diseases and conditions [9]. This document, developed jointly by the Academy of Osseointegration and the American Academy of Periodontology, endorses structured risk assessment at the time of implant planning, the integration of prosthetic design into peri-implant health preservation, and the implementation of individualised supportive care protocols, principles that are central to Statements 1, 5 and 6 of the present consensus. The convergence of the present recommendations with the EFP S3 guideline [8], the 2017 World Workshop classification [18], the ITI 6th Consensus [34] and the AO/AAP 2025 Consensus [9] underscores the international methodological consistency of the clinical questions addressed, while confirming that the evidence base underlying modern full-arch implant rehabilitation has reached a sufficient level of maturity to support evidence-based consensus formulation. Key methodological strengths include: (1) a multidisciplinary panel encompassing implant surgeons, prosthodontists, a dental hygienist with expertise in periodontal medicine, and dental technicians; (2) 100% participation rate across all three Delphi rounds; (3) a stringent consensus threshold (≥90%); and (4) transparent GRADE assessment for each statement. The inclusion of a dental hygienist reflects the growing recognition that long-term implant success depends not only on surgical and prosthetic factors, but also on structured preventive care delivered by the entire dental team. The translational contribution of the present document is the operationalisation of these internationally endorsed principles into a coherent and locally applicable clinical pathway, integrating surgical, prosthetic, biomechanical and maintenance decisions within a single risk-stratified framework (Table 3). National consensus documents serve a function complementary to international guidelines: while the latter establish the evidence-based core of recommendations, the former translate these into operational pathways adapted to defined healthcare settings, workforce competencies and reimbursement structures. The present consensus is positioned within this translational function and does not replace, but implements, the existing international frameworks.

### 4.1. Limitations

Several limitations should be acknowledged. First, evidence heterogeneity across the included studies precluded formal meta-analysis for some clinical questions. Second, the panel was composed predominantly of Italian clinicians and academics, which may limit generalizability to healthcare settings with different resources or patient populations. Third, the evidence base for some recommendations—particularly prosthetic materials (Statement 5)—relies primarily on short- to mid-term follow-up data; long-term outcomes beyond 10 years remain scarce. Fourth, patient-reported outcome measures (PROMs) were not systematically addressed, representing a gap in the current recommendations. Fifth, cost-effectiveness considerations were beyond the scope of this consensus. Sixth, although Statement 7 addresses systemic risk stratification and contraindications, the panel acknowledges that evidence on full-arch implant outcomes in systemically compromised patients (including those with uncontrolled diabetes, immunosuppression, or a history of head and neck radiotherapy) remains limited, and dedicated prospective studies in these populations are needed.

### 4.2. Clinical Implications

The present recommendations are intended to support—not replace—individualized clinical decision-making. Clinicians should interpret these statements within the context of patient-specific factors, available resources, and local healthcare settings. The emphasis on risk stratification throughout the document (Statements 1, 3, 6 and 7) reflects the panel’s conviction that standardized protocols must be adapted to individual patient profiles to optimize outcomes.

### 4.3. Future Research Priorities

The panel identifies the following priorities for future investigation: (1) long-term prospective studies (>15 years) comparing four- versus six-implant configurations in both arches; (2) randomized controlled trials comparing monolithic zirconia with metal-acrylic frameworks; (3) evaluation of digital workflows for surgical planning and prosthetic fabrication; (4) integration of patient-reported outcome measures into full-arch rehabilitation research; and (5) cost-effectiveness analyses to inform healthcare policy and resource allocation.

## 5. Conclusions

This consensus provides evidence-based recommendations for full-arch implant rehabilitation. Key findings include: (1) individualized periodontal risk assessment using validated tools; (2) four mandibular implants provide equivalent outcomes to six-implant configurations; (3) guided bone regeneration achieves predictable outcomes; (4) tilted and axial implants demonstrate equivalent performance; (5) monolithic zirconia is the material of choice; and (6) risk-stratified maintenance intervals reduce peri-implantitis by 75%.

## Figures and Tables

**Table 1:** Summary of Consensus Statements.

**Statement**	**Topic**	**Consensus (%)**	**GRADE**	**Strength**
1	Periodontal Risk Assessment	96	High	Strong
2	Guided Bone Regeneration	90	Moderate	Strong *
3	Number of Implants (Mandible)	94	High	Strong
4	Tilted vs. Axial Implants	94	High	Strong
5	Prosthetic Materials	91	High	Strong
6	Maintenance Protocols	98	High	Strong
7	Systemic Risk Stratification, Contraindications, and Perioperative Management	94	Moderate	Strong

* Strong recommendation despite moderate certainty of evidence, based on consistency of findings across studies, clear benefit-harm balance, and high clinical applicability (see Statement 2 rationale). Full-arch implant-supported rehabilitations should be considered in patients with a history of treated periodontitis only when periodontal stability has been clinically documented for a minimum of 6 months. Prior to surgical intervention, patients must undergo structured periodontal risk stratification using validated assessment tools. Implant therapy should be integrated into an individualized supportive periodontal and peri-implant maintenance program, as detailed in Statement 6.

**Table 2:** Risk-Based Maintenance Decision Matrix.

**Risk Category**	**Recall Interval**	**Mandatory Assessments**	**Intervention Thresholds**
Low: IDRA low; PRA low; Grade A; non-smoker; no systemic risk; cleanable prosthetic design	6 months	Peri-implant probing (6 sites/implant); bleeding on probing; full-mouth plaque score; annual periapical or panoramic radiograph; occlusal screening	BoP < 20% of sites: reinforce home care; no escalation. BoP ≥ 20% of sites or PD increase ≥ 2 mm at any implant: escalate to moderate-risk recall and initiate non-surgical intervention (mechanical debridement with antiseptic adjunct) in accordance with the EFP S3 peri-implant guideline.
Moderate: IDRA moderate; PRA moderate; Grade B; treated periodontitis; ex-smoker; controlled diabetes (HbA1c < 7.5%)	4 months	As above, with addition of: standardised photographic record; periapical radiographs every 12 to 18 months; STAB-based bruxism re-screening	BoP ≥ 30% of sites, or PD ≥ 5 mm with BoP at one or more implants: full peri-implant decontamination, antiseptic adjunct, re-evaluation at 2 months. Escalate to high-risk protocol if no resolution.
High: IDRA high; Grade C; active or untreated periodontitis; current smoker; uncontrolled diabetes (HbA1c ≥ 7.5%); definite bruxism; non-cleanable prosthetic design; poor compliance with supportive therapy	3 months	As above, with addition of: photographic record at every visit; periapical radiographs every 6 to 12 months; bruxism reassessment with portable electromyography when indicated; quantification of MBL relative to baseline	BoP ≥ 30% of sites, or PD ≥ 5 mm with BoP at one or more implants, or radiographic MBL ≥ 2 mm beyond baseline remodelling: surgical re-evaluation. Prosthesis removal is considered only when non-invasive assessment is precluded by prosthetic design.

Abbreviations: IDRA = Implant Disease Risk Assessment; PRA = Periodontal Risk Assessment; BoP = bleeding on probing; PD = probing depth; MBL = marginal bone loss; STAB = Standardised Tool for the Assessment of Bruxism; HbA1c = glycated haemoglobin.

**Table 3:** Operational Decision Algorithm for Full-Arch Implant Rehabilitation.

**Phase**	**Decision/Action**	**Operational Criteria and Statement Reference**
0	Systemic risk stratification: contraindications, MRONJ risk, and perioperative antibiotic prophylaxis	Structured assessment of ASA class, pharmacological history (bone-modifying agents, antiangiogenics, immunosuppressants, corticosteroids), comorbidities (diabetes, autoimmune disease), smoking status, and head-and-neck radiotherapy history. Absolute contraindications: HD-BMA R+/R++ for oncological indications, head-and-neck radiotherapy ≥ 60 Gy within 12 months, acute severe immunosuppression, uncontrolled bleeding disorders. LD-BMA Rx (osteoporosis): feasible with multidisciplinary consultation, elimination of local infectious foci, and written informed consent documenting non-quantifiable MRONJ risk. Antibiotic prophylaxis: chlorhexidine 0.20% pre- and post-operatively; amoxicillin 2 to 3 g single pre-operative dose; post-operative extension (up to 6 days) only when concomitant GBR or sinus elevation is performed; otherwise individualised based on patient risk and surgical complexity; clindamycin 600 mg in penicillin allergy. Per IAO 2021 and [51]. (Statement 7. Section 3.6)
1	Patient selection: periodontal stability or biological readiness	Partially edentulous: FMBS < 15%, FMPS < 20%, no PD ≥ 5 mm with BoP, no radiographic progression for ≥6 months. Fully edentulous: smoking cessation, HbA1c < 7.5%, prior SPT compliance, residual mucosal phenotype assessed. Susceptibility profiling by PRA + Staging/Grading + IDRA. (Statement 1; Section 2.4)
2	Surgical planning: prosthetically driven 3D planning with CAIS	CBCT and intraoral scan integrated into digital plan. Fully guided static CAIS is the standard of care for full-arch cases; dynamic CAIS an acceptable alternative. Freehand placement is not recommended. (Statement 4; Section 3.3.5)
3	Site preparation: bone augmentation only when prosthetically necessary	Evaluate tilted and short implants before indicating GBR. When GBR is required: one-stage approach if primary stability ≥ 35 Ncm or ISQ ≥ 70 and defect ≤ 2 mm; two-stage approach otherwise or in compromised wound-healing profiles. (Statement 2; Section 3.1.4)
4	Implant configuration: mandibular 4-implant baseline, 6 implants if high biomechanical risk	Six implants when: definite bruxism, severe atrophy, uncontrolled diabetes, full opposing fixed dentition, A-P spread < 5 mm, or when minimum individual fixture geometry cannot be achieved. (Statement 3. Section 3.3.6)
5	Implant features: length, diameter, and connection type	Terminal implants in four-implant mandibular designs: length ≥ 10 mm, diameter ≥ 4 mm. Conical implant-abutment connection (Morse-taper or platform-switched) preferred for crestal bone preservation and micromovement control. (Statement 4; Section 3.2.5, Section 3.3.6)
6	Loading protocol: immediate versus delayed function	Immediate function acceptable when insertion torque ≥ 35 Ncm or ISQ ≥ 70 at all implants; cross-arch splinting mandatory; avoid in definite bruxism with natural antagonist dentition. (Statement 4)
7	Prosthetic material and cantilever design: monolithic zirconia as first choice	Monolithic zirconia as first-choice definitive material; veneered zirconia discouraged in high-load profiles due to chipping risk. Cantilever length ≤ 1.5 × A-P spread; maximum 15 mm mandibular, 10 mm maxillary. Absolute contraindications to cantilever: definite bruxism with natural antagonist, prosthetic vertical dimension < 10 mm, A-P spread < 5 mm. (Statement 5; Section 3.3.7, Section 3.4.4)
8	Maintenance: risk-stratified recall and phased non-surgical and surgical intervention per EFP S3 peri-implant guideline	Low risk: 6 months; moderate risk: 4 months; high risk: 3 months. Phased non-surgical and surgical intervention applied when BoP and PD thresholds reached, per EFP S3 peri-implant guideline. Lifelong follow-up. (Statement 6; Section 3.5, Table 2)

Abbreviations: ASA = American Society of Anesthesiologists physical status classification; HD-BMA = high-dose bone-modifying agent; LD-BMA = low-dose bone-modifying agent; MRONJ = medication-related osteonecrosis of the jaw; IAO = Italian Academy of Osseointegration; CBCT = cone-beam computed tomography; CAIS = computer-aided implant surgery (s-CAIS = static; d-CAIS = dynamic); GBR = guided bone regeneration; ISQ = implant stability quotient; A-P = anteroposterior; BoP = bleeding on probing; PD = probing depth; HbA1c = glycated haemoglobin; IDRA = Implant Disease Risk Assessment; PRA = Periodontal Risk Assessment; FMBS = full-mouth bleeding score; FMPS = full-mouth plaque score; SPT = supportive periodontal therapy.

## Data Availability

Not applicable.

## References

[1] Feine J.S., Carlsson G.E., Awad M.A., Chehade A., Duncan W.J., Gizani S., Head T., Heydecke G., Lund J.P., MacEntee M. (2002). The McGill consensus statement on overdentures. Mandibular two-implant overdentures as first choice standard of care for edentulous patients. Gerodontology.

[2] British Society for the Study of Prosthetic Dentistry (2009). The York consensus statement on implant-supported overdentures. Eur. J. Prosthodont. Restor. Dent..

[3] Maló P., de Araújo Nobre M., Lopes A., Ferro A., Nunes M. (2019). All-on-4 Concept: A Longitudinal Study with 5–13 Years of Follow-up. Clin. Implant Dent. Relat. Res..

[4] Sharaf M.A., Wang S., Mashrah M.A., Xu Y., Haider O., He F. (2024). Outcomes that may affect implant and prosthesis survival and complications in maxillary fixed prosthesis supported by four or six implants: A systematic review and meta-analysis. Heliyon.

[5] Malak A.A., El Masri Y., El Masri J., Issawi H.A., Salameh P., Aoun G. (2024). Implant Failure and Marginal Bone Loss Between Axial and Tilted Implants: An Umbrella Review with Meta-analysis. Int. J. Oral Maxillofac. Implant..

[6] AlTarawneh S., Thalji G., Cooper L. (2021). Full-arch implant-supported monolithic zirconia fixed dental prostheses: An updated systematic review. Int. J. Oral Implantol..

[7] Papaspyridakos P., Sinada N., Ntovas P., Barmak A.B., Chochlidakis K. (2025). Zirconia full-arch implant prostheses: Survival, complications, and prosthetic space dimensions with 115 edentulous jaws. J. Prosthodont..

[8] Herrera D., Berglundh T., Schwarz F., Chapple I., Jepsen S., Sculean A., Kebschull M., Papapanou P.N., Tonetti M.S., Sanz M. (2023). EFP workshop participants and methodological consultant. Prevention and treatment of peri-implant diseases—The EFP S3 level clinical practice guideline. J. Clin. Periodontol..

[9] Wang H.L., Avila-Ortiz G., Monje A., Kumar P., Calatrava J., Aghaloo T., Barootchi S., Fiorellini J.P., Galarraga-Vinueza M.E., Kan J. (2025). AO/AAP consensus on prevention and management of peri-implant diseases and conditions: Summary report. J. Periodontol..

[10] Ruiz-Romero V., Figueiredo R., Toledano-Serrabona J., Abdelazim Y., Camps-Font O., Salazar-Salazar Y., Plana-Soler A., Subirà-Pifarré C., Valmaseda-Castellón E. (2024). Peri-implantitis in patients without regular supportive therapy: Prevalence and risk indicators. Clin. Oral Investig..

[11] Brouwers M.C., Kho M.E., Browman G.P., Burgers J.S., Cluzeau F., Feder G., Fervers B., Graham I.D., Grimshaw J., Hanna S.E. (2010). AGREE II: Advancing guideline development, reporting and evaluation in health care. CMAJ.

[12] Guyatt G., Oxman A.D., Akl E.A., Kunz R., Vist G., Brozek J., Norris S., Falck-Ytter Y., Glasziou P., DeBeer H. (2011). GRADE guidelines: 1. Introduction—GRADE evidence profiles and summary of findings tables. J. Clin. Epidemiol..

[13] Lang N.P., Tonetti M.S. (2003). Periodontal risk assessment (PRA) for patients in supportive periodontal therapy (SPT). Oral Health Prev. Dent..

[14] Tonetti M.S., Greenwell H., Kornman K.S. (2018). Staging and grading of periodontitis: Framework and proposal of a new classification and case definition. J. Periodontol..

[15] Serroni M., Borgnakke W.S., Romano L., Balice G., Paolantonio M., Saleh M.H.A., Ravidà A. (2024). History of periodontitis as a risk factor for implant failure and incidence of peri-implantitis: A systematic review, meta-analysis, and trial sequential analysis of prospective cohort studies. Clin. Implant Dent. Relat. Res..

[16] Roccuzzo A., Imber J.C., Marruganti C., Salvi G.E., Ramieri G., Roccuzzo M. (2022). Clinical outcomes of dental implants in patients with and without history of periodontitis: A 20-year prospective study. J. Clin. Periodontol..

[17] Sanz M., Herrera D., Kebschull M., Chapple I., Jepsen S., Beglundh T., Sculean A., Tonetti M.S., EFP Workshop Participants and Methodological Consultants (2020). Treatment of stage I-III periodontitis—The EFP S3 level clinical practice guideline. J. Clin. Periodontol..

[18] Berglundh T., Armitage G., Araujo M.G., Avila-Ortiz G., Blanco J., Camargo P.M., Chen S., Cochran D., Derks J., Figuero E. (2018). Peri-implant diseases and conditions: Consensus report of workgroup 4 of the 2017 World Workshop on the Classification of Periodontal and Peri-Implant Diseases and Conditions. J. Clin. Periodontol..

[19] Heitz-Mayfield L.J.A., Heitz F., Lang N.P. (2020). Implant Disease Risk Assessment IDRA—A tool for preventing peri-implant disease. Clin. Oral Implant. Res..

[20] De Ry S.P., Roccuzzo A., Lang N.P., Heitz-Mayfield L.J., Ramseier C.A., Sculean A., Salvi G.E. (2021). Evaluation of the implant disease risk assessment (IDRA) tool: A retrospective study in patients with treated periodontitis and implant-supported fixed dental prostheses (FDPs). Clin. Oral Implant. Res..

[21] Elnayef B., Porta C., del Amo F.S.L., Mordini L., Gargallo-Albiol J., Hernández-Alfaro F. (2018). The Fate of Lateral Ridge Augmentation: A Systematic Review and Meta-Analysis. Int. J. Oral Maxillofac. Implant..

[22] Urban I.A., Monje A. (2000). Guided Bone Regeneration in Alveolar Ridge Reconstruction. Periodontology.

[23] Monje A., Pons R., Insua A., Nart J., Wang H.L., Schwarz F. (2019). Morphological and Clinical Complications in Guided Bone Regeneration Procedures: A Systematic Review. Clin. Oral Implant. Res..

[24] Esposito M., Grusovin M.G., Felice P., Worthington H.V. (2014). Interventions for replacing missing teeth: Different types of dental implants. Cochrane Database Syst. Rev..

[25] Thoma D.S., Zeltner M., Hüsler J., Hämmerle C.H.F., Jung R.E. (2015). EAO Consensus: Short implants versus augmentation procedures. Clin. Oral Implant. Res..

[26] Maló P., de Araújo Nobre M., Lopes A., Ferro A., Gravito I. (2011). All-on-4 concept for full-arch rehabilitation: Avoiding bone grafting. Clin. Implant Dent. Relat. Res..

[27] Caramês J.M.M., Francisco H.C.O., Vieira F.A., Caramês G.B., Martins J.N.d.R., Marques D.N.d.S. (2025). Four vs. Six Implant Full-Arch Restorations—A Direct Comparative Retrospective Analysis in a Large Controlled Treatment Cohort. J. Clin. Med..

[28] Bevilacqua M., Tealdo T., Menini M., Pera F., Mossolov A., Drago C., Pera P. (2011). The influence of cantilever length and implant inclination on stress distribution in maxillary implant-supported fixed dentures. J. Prosthet. Dent..

[29] Chrcanovic B.R., Albrektsson T., Wennerberg A. (2015). Tilted versus axially placed dental implants: A meta-analysis. J. Dent..

[30] Mehta S.P., Sutariya P.V., Pathan M.R., Upadhyay H.H., Patel S.R., Kantharia N.D.G. (2021). Clinical success between tilted and axial implants in edentulous maxilla: A systematic review and meta-analysis. J. Indian Prosthodont. Soc..

[31] Vercruyssen M., Laleman I., Jacobs R., Quirynen M. (2015). Computer-supported implant planning and guided surgery: A narrative review. Clin. Oral Implant. Res..

[32] Degidi M., Daprile G., Piattelli A. (2012). Primary stability determination by means of insertion torque and RFA in a sample of 4,135 implants. Clin. Implant Dent. Relat. Res..

[33] Cinquini C., Alfonsi F., Marchio V., Gallo F., Zingari F., Bolzoni A.R., Romeggio S., Barone A. (2023). The Use ofZirconia for Implant-Supported Fixed Complete Dental Prostheses: A Narrative Review. Dent. J..

[34] Morton D., Gallucci G., Lin W.S., Pjetursson B., Polido W., Roehling S., Sailer I., Aghaloo T., Albera H., Bohner L. (2018). Group 2 ITI Consensus Report: Prosthodontics and implant dentistry. Clin. Oral Implant. Res..

[35] Lin W.S., Eckert S.E. (2018). Clinical performance of intentionally tilted implants versus axially positioned implants: A systematic review. Clin. Oral Implant. Res..

[36] Khaohoen A., Powcharoen W., Sornsuwan T., Chaijareenont P., Rungsiyakull C., Rungsiyakull P. (2024). Accuracy of implant placement with computer-aided static, dynamic, and robot-assisted surgery: A systematic review and meta-analysis of clinical trials. BMC Oral Health.

[37] Lau K., Fung T.K.C., Ho D.K.L., Pelekos G., Fok M.R. (2025). Accuracy of static and dynamic computer-aided implant surgery for immediate implant placement: A systematic review and meta-analysis. J. Prosthodont. Res..

[38] Macedo J.P., Pereira J., Vahey B.R., Henriques B., Benfatti C.A.M., Magini R.S., López-López J., Souza J.C.M. (2022). Comparison of external, internal flat-to-flat, and conical implant abutment connections for implant-supported prostheses: A systematic review and network meta-analysis of randomized clinical trials. J. Prosthet. Dent..

[39] Caricasulo R., Malchiodi L., Ghensi P., Fantozzi G., Cucchi A. (2018). The influence of implant-abutment connection to peri-implant bone loss: A systematic review and meta-analysis. Clin. Implant Dent. Relat. Res..

[40] Schoenbaum T.R., Karateew E.D., Schmidt A., Jadsadakraisorn C., Neugebauer J., Stanford C.M. (2023). Implant-Abutment Connections and Their Effect on Implant Survival Rates and Changes in Marginal Bone Levels (ΔMBL): A Systematic Review and Meta-Analysis of 45,347 Oral Implants. Int. J. Oral Maxillofac. Implant..

[41] Lobbezoo F., Ahlberg J., Raphael K.G., Wetselaar P., Glaros A.G., Kato T., Santiago V., Winocur E., De Laat A., De Leeuw R. (2018). International consensus on the assessment of bruxism: Report of a work in progress. J. Oral Rehabil..

[42] Verhoeff M.C., Lobbezoo F., Ahlberg J., Bender S., Bracci A., Colonna A., Dal Fabbro C., Durham J., Glaros A.G., Häggman-Henrikson B. (2025). Updating the Bruxism Definitions: Report of an International Consensus Meeting. J. Oral Rehabil..

[43] Manfredini D., Ahlberg J., Aarab G., Bracci A., Durham J., Ettlin D., Gallo L.M., Koutris M., Wetselaar P., Svensson P. (2020). Towards a Standardised Tool for the Assessment of Bruxism (STAB)—Overview and general remarks of a multidimensional bruxism evaluation system. J. Oral Rehabil..

[44] Häggman-Henrikson B., Ali D., Aljamal M., Chrcanovic B.R. (2024). Bruxism and dental implants: A systematic review and meta-analysis. J. Oral Rehabil..

[45] Monje A., Aranda L., Diaz K.T., Alarcón M.A., Bagramian R.A., Wang H.L., Catena A. (2016). Impact of Maintenance Therapy for the Prevention of Peri-implant Diseases: A Systematic Review and Meta-analysis. J. Dent. Res..

[46] Bedogni A., Mauceri R., Fusco V., Bertoldo F., Bettini G., Di Fede O., Lo Casto A., Marchetti C., Panzarella V., Saia G. (2024). Italian position paper (SIPMO-SICMF) on medication-related osteonecrosis of the jaw (MRONJ). Oral Dis..

[47] Ruggiero S.L., Dodson T.B., Aghaloo T., Carlson E.R., Ward B.B., Kademani D. (2022). American Association of Oral and Maxillofacial Surgeons’ Position Paper on Medication-Related Osteonecrosis of the Jaws—2022 Update. J. Oral Maxillofac. Surg..

[48] Campisi G., Mauceri R., Bertoldo F., Bettini G., Biasotto M., Compilato D., Di Fede O., Bedogni A. (2022). The preventive care of medication-related osteonecrosis of the jaw (MRONJ): A position paper by Italian experts for dental hygienists. Support. Care Cancer.

[49] Caiazzo A., Canullo L., Pesce P., Consensus Meeting Group (2021). Consensus Report by the Italian Academy of Osseointegration on the Use of Antibiotics and Antiseptic Agents in Implant Surgery. Int. J. Oral Maxillofac. Implant..

[50] Romandini M., De Tullio I., Congedi F., Kalemaj Z., D’Ambrosio M., Laforí A., Quaranta C., Buti J., Perfetti G. (2019). Antibiotic prophylaxis at dental implant placement: Which is the best protocol? A systematic review and network meta-analysis. J. Clin. Periodontol..

[51] Momand P., Naimi-Akbar A., Hultin M., Lund B., Götrick B. (2024). Is routine antibiotic prophylaxis warranted in dental implant surgery to prevent early implant failure?—A systematic review. BMC Oral Health.

